# Biological Parameters, Phenology and Temperature Requirements of *Halyomorpha halys* (Hemiptera: Pentatomidae) in the Sub-Mediterranean Climate of Western Slovenia

**DOI:** 10.3390/insects13100956

**Published:** 2022-10-19

**Authors:** Mojca Rot, Lara Maistrello, Elena Costi, Stanislav Trdan

**Affiliations:** 1 Institute of Agriculture and Forestry Nova Gorica, Pri hrastu 18, 5000 Nova Gorica, Slovenia; 2 Department of Agronomy, Biotechnical Faculty, University of Ljubljana, 1000 Ljubljana, Slovenia; 3 Dipartimento di Scienze della Vita, Università di Modena e Reggio Emilia, 42122 Reggio Emilia, Italy

**Keywords:** *Halyomorpha halys*, phenology, temperature requirements, voltinism, life table

## Abstract

**Simple Summary:**

The invasive brown marmorated stink bug *Halyomorpha halys*, native to East Asia, has become one of the most damaging agricultural pests worldwide. After being first detected in Europe (in Switzerland), it is now widely spread throughout the European continent and many countries in Eurasia. Since its first appearance in Slovenia in 2017, it has caused extensive damage to fruit and vegetable production. Investigating the biology and behavior in local environmental conditions is the first step towards effective pest control. Information on the number of generations per year is crucial for anticipating critical phases of pest development and for adapting control measures that target the pest’s vulnerable life stages. A 3-year study (2019–2021) on the biological parameters of *H. halys* was performed outdoors in Nova Gorica (western Slovenia), confirming that in the sub-Mediterranean climate this pest has two overlapping generations per year. The net reproductive rates observed over the period studied indicate growing populations. The highest population growth was recorded in 2019, when the net reproductive rate of increase (R0) reached 14.84 for the summer generation and 5.64 for the overwintering generation. These findings reflect the current situation in Slovenia, where the growing populations of *H. halys* has been causing considerable damage to agricultural crops since 2019.

**Abstract:**

In the last decade, the invasive brown marmorated stink bug *Halyomorpha halys*, native to East Asia, has become one of the most serious pests for agricultural crops worldwide. First detected in Europe (in Switzerland), the insect is now widely found across the European continent and many Eurasian countries. Since its first appearance in Slovenia in 2017 it has caused considerable damage to fruit and vegetable production. Understanding the biology and behavior in the local environmental conditions is of key importance for an effective pest management. Knowledge of the voltinism of the species is crucial to anticipate critical phases of pest development and for adapting control measures that target the vulnerable life stages of the pest. A 3-year study (2019–2021) of *H. halys* biological parameters was performed outdoors in Nova Gorica (western Slovenia), confirming that in the sub-Mediterranean climate this pest has two overlapping generations per year. The net reproductive rates observed in the studied period indicate growing populations. The highest population growth was recorded in 2019, when the net reproductive rate of increase (R_0_) reached 14.84 for the summer generation and 5.64 for the overwintering generation. These findings match the current situation in Slovenia, where increasing populations of *H. halys* and severe crop damage have been observed since 2019.

## 1. Introduction

*Halyomorpha halys* (Stål, 1855) (Heteroptera, Pentatomidae), also known as the brown marmorated stink bug, is native to East Asia and has rapidly become a globally distributed invasive species by stowing away on traded goods and people traveling [1]. Accidental multiple introductions and spread of *H. halys* outside its native range were first reported in North America [2,3] and central–southern Europe [4,5] followed by the subsequent invasion in Eurasia [6], South America [7] and North Africa [8]. This polyphagous pest with an extremely wide host range [9] has a high capacity to cause damage to agricultural production. Significant losses in pome and stone fruit, hazelnuts, vegetables and row crops from several countries have been reported during the outbreak years. The United States were the first to suffer severe agricultural damage following the introduction of *H. halys* [10], resulting in loss of USD 37 million in apple production [11] and severe damage also in peach orchards in 2010 in the mid-Atlantic region. Damage to sweet corn, soybean, beans, peppers and tomatoes have also been reported in the same region [12]. In Europe, Italy is the country most affected by this invasive species. A few years after its first discovery, in 2015, *H. halys* became the main key pest of fruit orchards in northern Italy [13], with the greatest damage especially in pears [14]. It also caused severe damage to hazelnut production in 2017 [15]. Losses in fruit production in northern Italy peaked in 2019 when the estimated damage was nearly EUR 600 million [16]. Since 2015, *H. halys* has affected agricultural production in many other European and Asian countries. Economic losses have been reported in orchards in the southern and northeastern Switzerland [17], in vegetable crops in Hungary [18], in peaches and olives in Greece [19], in fruit crops in Russia, Abkhazia and Serbia [20], and in hazelnuts in Georgia, where it caused USD 60 million damage [21]. *Halyomorpha halys* was first detected in Slovenia in 2017 [22], where it caused considerable damage in peach, pear and apple orchards, mainly in western Slovenia [23].

Monitoring of the global invasion of *H. halys* indicates that the degree of damage to crops is closely related to the phenology and voltinism of the pest. Abiotic conditions during the growing season affect the number of generations produced per year by multivoltine insects like *H. halys*, which in turn can affect crop losses [24]. In newly invaded areas with favorable environmental conditions, which allow the development of two generations per year, severe damage was observed in less than three years after *H. halys* establishment. Italy and Georgia are good examples of this [13,15].

Insect phenology is primarily driven by temperature and photoperiod [25,26]. Recent research has shown that *H. halys* development, survival, voltinism, population density and size, as well as overwintering behavior, depend on temperature and photoperiod, which can vary with latitude and other geographical parameters [27,28,29,30]. In its native range, in northern China, Korea and Japan, *H. halys* is univoltine or bivoltine [31]. In the northern latitudes of the USA in Pennsylvania, New Jersey and New York it develops one generation per year [27,32], while in the southern states of West Virginia, Virginia, North Carolina and California, two generations per year occur [33,34,35]. In northern Switzerland, *H. halys* is univoltine [29], in northern Italy [30] as well as in the subtropical climate zone of western Georgia it is bivoltine [21].

Understanding the biology and phenology of new invasive pest species in a given geographic area, under local climatic conditions is the first step in developing an effective pest control strategy. As Slovenia is a topographically as well as climatically very diverse country, it is difficult to predict the new pest behavior in its invaded habitat in Slovenia on the basis of foreign research data alone. Therefore, the main objective of this work was to investigate biological parameters and behavior of *H. halys* in the sub-Mediterranean climate of western Slovenia and to gain new knowledge that helps optimize pest control strategies and prevent damage to crops in the country.

## 2. Material and Methods

### 2.1. Phenology, Lifetime Fecundity and Mortality Assessment in Outdoor Conditions

The biological characteristics of *H. halys* were studied systematically from 2019 to 2021 at the Institute of Agriculture and Forestry in Nova Gorica (45°57′39.32″ N, 13°39′30.72″ E), western Slovenia under outdoor temperature and humidity conditions, protected from rain and direct sunlight. Outdoor temperatures were recorded with a data logger (Voltcraft DL-210TH). In autumn 2018, adult stink bugs were collected from wild and cultivated host plants in the nearby gardens and orchards and outside the buildings in the vicinity of Nova Gorica. The same protocol described by Costi et al. [30] was adopted to obtain the overwintering survival rate of adults. Three hundred adult bugs (150 females, 150 males) were placed in a winter shelter made of a wooden box (20 × 30 × 20 cm) with a thickness of 1.5 cm on the sides and filled with cardboard sheets. A 5 mm wide slit was made on the top of the box to allow adults to perceive the photoperiod and exit outside the box in spring. To prevent adults from escaping after the spring exit, the wooden box was placed inside a large plastic box with mesh side windows. From the beginning of January onwards, the number of adults coming out of the wooden box was monitored every 2 days. The adults were counted, sexed and marked with color in order to separate them from the newly emerged ones. The overwintering survival rate was evaluated 2 weeks after the last adults emerged.

The successfully surviving specimens were transferred to ventilated plastic box (47 × 35 × 25 cm) with a solid bottom and mesh windows on all sides (rearing box). The rearing diet adopted according to Funayama and Medal et al. [36,37] consisted of green beans, a mixture of oil seeds (sunflower and pumpkin seeds), peanuts and pieces of carrots. An ornamental plant (*Peperomia obtusifolia* Variegata) was provided as a shelter and also as food source. Two water-soaked cotton balls, 5 cm in diameter, were added in the box. Food and water were changed twice a week.

After the first mating was observed, 10 females and 10 males were randomly selected from the rearing box and transferred together to a smaller ventilated plastic box (15 × 18 × 10 cm). Food and water were provided as previously described. A piece of green paper (6 × 9 cm) was added to the box as oviposition substrate. Adult pairs were monitored daily for egg-laying and mortality. Any egg mass laid was removed from the box and the eggs were counted. The first four egg masses laid in each week were selected for the development follow-up. The number of hatched eggs, the number of first instar nymphs and mortality rates were recorded. After the first molt, ten 2nd instar nymphs were randomly selected from each egg mass, moved to ventilated plastic containers (10 × 10 × 10 cm) and their development was followed daily until the emergence of the adult stage. Development time and mortality were recorded for each instar. The same procedure was implemented for the summer generation. Ten females and ten males were randomly selected from the newly emerged adult population and data on their offspring was recorded until reaching adulthood. Overall, *H. halys* population was studied for 3 years (2019–2021) following the same procedure described above.

### 2.2. Life-Tables Analyses

Standard methods [29,30] were used to construct life tables of *H. halys* for the two separate generations studied in 2019. To estimate generational mortality and to examine mortality in all life stages, life tables were built on the basis of data obtained in the previously described experiments. A group of egg masses and individual nymphs of all five instars (N1–N5; N = 40) were selected weekly and then followed up to adulthood, and they were the only representatives for each cohort. The actual mortality data for eggs and nymphs observed in the study were first transformed into proportional data and then applied to the actual number of eggs laid during each time period (cohort).

Main measures to express mortality according to Bellows et al. [38] were applied in the life tables, as follows: 

Apparent mortality (q_x_) = [d_x_/l_x_] was calculated as a fraction of died individuals in a specific stage (d_x_) out of the total number of individuals entered in the same stage (l_x_).

Real mortality (r_x_) = [dx/l_0_] was calculated as a fraction of died individuals in a specific stage (d_x_) out of the total number initially entering the first stage in a life table (l_0_).

k-values (k_x_) = −log (1 − m_x_): the k_x_-values which express the intensity of mortality are the negative logarithm of (1-marginal attack rate (mx)) for a factor. When only one mortality factor occurs in a stage, or where more than one occurs and they act sequentially, then the apparent mortality (q_x_) equals the marginal attack rate (m_x_).

Generation mortality (K_s_) is the sum of all mortality factor k-values and represents the total mortality for the generation (summed over all life stages). K_s_ = k_eggs_ + k_N1_ + k_N2_ + k_N3_ + k_N4_ + k_N5._

The proportion of the total generational mortality 100k_x_/K_s_: this value shows the impact of mortality in each stage on the generational mortality of *H. halys*.

The net reproductive rate of increase (R_0_), which describes the number of times the population increases or decreases from one generation to the next [39], was calculated for both the overwintering and the summer generation of *H. halys* according to the formula:

R_0 overwintering generation_ = realized progeny/number of eggs in the overwintering generation (l_0_ = 1724)

R_0 summer generation_ = realized progeny/number of eggs in the summer generation (l_0_ = 760)

The R_0_ values for growing populations are greater than 1, whereas values for declining populations are less than 1.

### 2.3. Data Analysis

The relationships between temperature (x) and the development time (y) were determined using a simple linear regression analysis (y = kx + n) [40]. Statistical analyses were performed using Statgraphics Centurion XVI software (Statgraphics Technologies Inc., The Plains, VA, USA) and the results are presented as untransformed mean ± standard error (SE).

## 3. Results

### 3.1. Overwintering Survival and Seasonal Phenology

The overwintering survival rate of adults was followed and assessed in the 2018/2019, 2019/2020 and 2020/2021 seasons. In all 3 years, some single specimens emerged from overwintering cages at the end of January and in the first week of February, respectively. However, they were unable to survive in cold weather conditions. In all 3 years, the noticeable adult emergence began at the end of March, when average daily temperatures exceeded 12 °C and peaked at the end of April, with average daily temperatures above 15 °C. Overwintering survival rates during the three following years were 39.9%, 40.3% and 36.5%, respectively. One third to one half of the adults who survived the winter died before the mating season. Total overwintering mortality of adults in the 3 years was 78.8%, 71.2%, and 80.8%. Oviposition began when average daily temperatures reached 17 °C, and the earliest was recorded in 11 May 2020. In the other 2 years, the first egg laying was observed at the end of May. The oviposition period of overwintering generations lasted 12–13 weeks. In 2020 and 2021 the last eggs were laid in the first half of August, while in 2019 the overwintering generation laid eggs from the end of May until the end of third week of August (Table 1). The oviposition period of the summer generation was shorter, as females laid eggs from late July to late August. 8 September was the latest date of oviposition recorded over the 3-year monitoring period (Table 2). The nymphs that emerged from the eggs laid in September have never reached adulthood.

### 3.2. Egg to Adult Development under a Natural Fluctuating Temperature Regime

**Figure 1:**
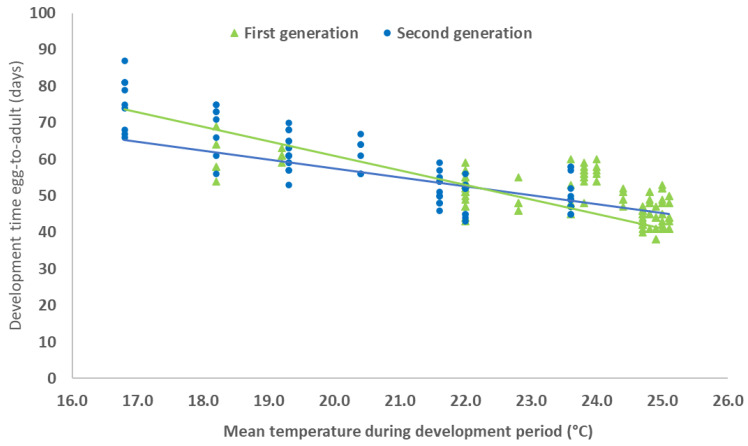
Duration (days) of *Halyomorpha halys* development from egg to adult as a function of temperatures during the development period at Nova Gorica (western Slovenia) in 2019; described by the linear model (y = −2.4475x + 106.49; R^2^ = 0.4568) for first generation (N = 200) and (y = −3.9649x + 140.23; R^2^ = 0.7129) for second generation (N = 73). Green triangles represent the eggs laid from 2 June to 22 August and developing to adults from mid of July to end of October. Blue dots represent the eggs laid from 30 July to 27 August and developing to adults from mid of September to beginning of November.

The time elapsed between the exit from overwintering and the first egg laying (i.e., pre-oviposition period for the overwintering females) was 39.43 ± 3.28 days. The shortest time was recorded in 2020, when females needed an average of 33.2 days at an average temperature of 15.9 °C during this period. The pre-oviposition period was generally shorter at high temperatures. Females of the summer generation required 14.0 ± 1.11 to 18.6 ± 0.58 days to start oviposition after their emergence as adults (Table 3), at mean daily temperatures of 23.8 to 24.8 °C. At naturally fluctuating temperatures, complete development from egg to adult lasted 52.24 ± 3.55 days in the overwintering generation and 55.89 ± 1.68 days in the summer generation. The shortest egg-to-adult development time (38 days) was recorded in the overwintering generation, at the average temperature of 25.0 °C during development period (Figure 1). The longest development time (81 days) was that of second generation adults, which developed from the eggs laid at the end of August 2019, at an average temperature of 16.8 °C during the development period.

**Figure 2:**
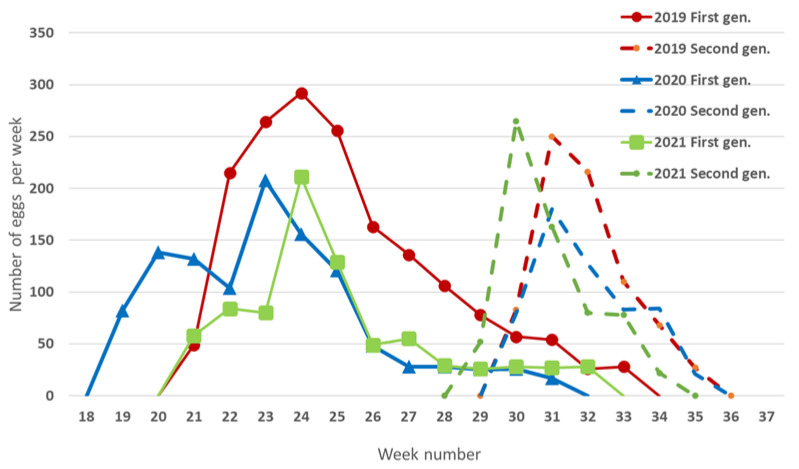
Halyomorpha halys overwintering and summer generation oviposition patterns over 3-year period (2019–2021) in Nova Gorica (western Slovenia). In all 3 years, 10 females were followed for the two generations.

**Figure 3:**
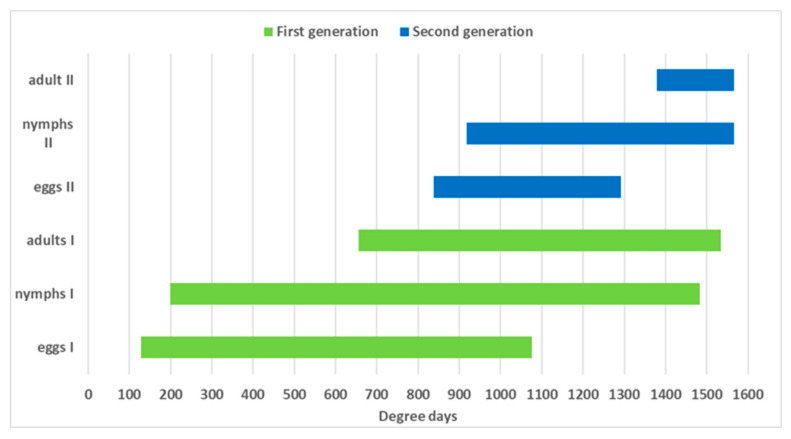
*Halyomorpha halys* phenology based on the average degree-day requirements for eggs, nymphs and adults of first and second generation, recorded at outdoor conditions in Nova Gorica (western Slovenia) in a period of 3 years (2019–2021).

The accumulation of degree days was calculated using the lower threshold of 12.2 °C [29] starting from 1 January. The average accumulation of degree days from adult emergence to first oviposition was 118.77 ± 7.72 degree-days over the 3-year period (Table 4). Oviposition of the summer generation began at 837.8 degree-days and peaked between 950 and 1050 degree-days (30–31 calendar week; from the end of July to beginning of August). The average degree-day requirement for egg-laying of the overwintering generation was 128.3 degree-days, with an oviposition peak between 250 and 400 degree-days; (23–25 calendar week; 10–20 June) (Figure 2). The nymphs of the first instar of the overwintering generation occurred at 200 degree-days. 20 May was the earliest date at which the N1 nymphs occurred in the 3-year period. The N1 nymphs of the summer generation occurred from late July until early August at around 917.8 degree-days (Figure 3). The mean number of degree-days required for egg-to-adult development in the 3-year period was 530.87 ± 12.40 for the first generation and 545.5 ± 16.61 for the second generation. The first adults of the first generation emerged in mid-July and the first adults of the second generation emerged in mid-September. In a 3-year period, the two generations had overlapped starting in early August, when the first nymphs of the second generation emerged.

The highest fecundity of our experimental populations was recorded in 2019, when overwintering couples (*n* = 10) produced in total 1742 eggs, with the average lifetime fecundity of 174.2 ± 19.07 eggs per female. The females of the summer generation (*n* = 10) laid in total 760 eggs; the average lifetime fecundity was 76.0 ± 11.73 per female. The females of the overwintering population laid 3–11 egg masses, while the females of the summer generation laid 1–6 egg masses. Mating and oviposition were not observed among newborn adults of the summer generation, and the development of the third generation did not occur.

The overall mortality in 2019 has reached different rates between development stages and generations within cohorts weakly. The average mortality of overwintering generation ranged from 5.0 to 22.4% in the overwintering generation (Table 1) and from 8.0 to 27.3% in the summer generation (Table 2). The highest mortality was recorded among second instar nymphs (22%) and in the egg stage (18%) in the overwintering generation, while in the summer generation the highest mortality was recorded among the second and third instar nymphs, where the mortality rates were 20 and 27%, respectively.

### 3.3. Life Table Parameters for Halyomorpha Halys Population in 2019

The total generation mortality of the summer generation (89.86%) (Table 5) was higher compared to that of the overwintering generation (60.94%) (Table 6). The mortality rate of diapausing adults reached 54.52% and contributed the most to the generational mortality, while the mortality during the second nymphal stage, with 27.81%, contributed the most to the total generational mortality of the overwintering generation. The net reproductive rate (R_0_) of the overwintering and summer generations were 14.84 and 5.64, respectively.

## 4. Discussion

As *H. halys* is a recently established invasive pest in Europe, there is still a lack of basic biological knowledge in the newly invaded areas, which is essential for the development of management solutions. Three studies of *H. halys* biology have been carried out in Europe so far, namely Swiss research that reported the existence of one generation/year in the area of Zurich [29], an Italian study showing that south of the Alps *H. halys* develops two generations/year [30] and Russian one which confirmed bivoltinism of the species in Sochi region [41]. The basic findings of our research are in line with the results of Italian study. In western Slovenia, *H. halys* developed two generations/year for 3 consecutive years. The favorable temperature conditions in Nova Gorica (western Slovenia) with monthly average temperatures 12 °C in April and 16.7 °C in May allowed for the early emergence of adults from the winter shelters and the early start of oviposition from mid-May to late May. Similarly, the early onset of oviposition has been reported in the province of Modena (Italy) [30], and in the Chinese province of Beijing [42], where *H. halys* populations are also bivoltine. Subsequently, the development of the nymphs was driven by the high summer temperatures of June and July, which accelerated the emergence of adults. The first adults of the summer generation appeared from mid-July onwards, which is also in good agreement with the Chinese and Italian results, where the new generation adults emerged in the beginning of July. In addition to temperature, which is the main driver of insect development, photoperiod is a crucial factor in determining the developmental pattern [43]. Haye et al. [29] suggested that only first-generation adults who developed under long day conditions (day length greater than 15 h) are able to produce the second generation. Taking this into account, only 30% of the adults of the first generation of our study were able to develop the second generation. The second generation adults did not occur before mid-September when the photoperiod was already below 13 h light. These adults showed no reproductive behavior and immediately entered diapause. This is consistent with findings of Costi et al. [30] and other studies that confirmed day length is the dominant environmental cue for *H. halys* entry into the reproductive diapause [32,44,45,46,47]. 

The overall overwintering mortality rates observed in 3-year period ranged from 71 to 81%, slightly lower than the results for northern Italy (86%), but much higher than those recorded in the Swiss survey (39%). Temperature is known to play a critical role in the biology of *H. halys* [48]. Being a cold-intolerant species, exposure to low temperatures during the winter and extreme spring frost events can strongly contribute to increase the total mortality of *H. halys* [49]. According to these authors, survival rates begin to decline when temperatures drop to −5 °C. The lowest winter temperature measured during our 3-year research was −5.3 °C and the lowest recorded spring temperature was −2.5 °C in early April 2021, when a severe frost wave hit the whole central Europe. In addition to cold winter temperatures and spring frost events, low nutritional level of early emerged adults is another important factor contributing to total overwintering mortality. Costi et al. [30] (2017) found that only half of all the successfully overwintered adults were able to survive and reach the reproductive stage. In the Goriška region (western Slovenia), the first adults emerged from winter shelters as early as late January, but were unable to survive. According to Funayama [50], adults that have not accumulated sufficient nutritional reserves tend to exit the diapause earlier. Since second-generation adults have a shorter period to accumulate nutrient reserves for overwintering, this is likely to be the reason why bivoltine populations of *H. halys* have higher overwintering mortality than univoltine populations [51]. This could also be the explanation for the much lower mortality rate of the univoltine population recorded in Switzerland compared to the Italian and Slovenian bivoltine populations.

The pre-oviposition period of *H. halys* in our study averaged 39.43 ± 3.28 days for the overwintering population with an average 174.2 ± 19.07 eggs laid per female and 16.63 ± 1.37 days for the summer generation, with an average 76.0 ± 11.73 SE eggs laid per female. Fecundity was much lower than that observed in the native area in Asia, where a single female can produce over 480 eggs in her lifetime [52] and even lower than the Italian data with lifetime fecundity of the overwintering generation and of the summer generation with 285 and 214 eggs per female, respectively [30]. The higher latitude, together with less favorable climatic conditions, in particular the temperature, resulted in a shorter egg-laying period in both generations. Slovenian overwintering generations started with the oviposition at the end of May, that is on average 2–3 weeks later than that was found for the Italian population [30]. Subsequently, this delays the appearance of summer generation adults, which begin to reproduce when the photoperiod is already decreasing. Our observations could be well explained by research of Reznik et al. [41], who found that with decreasing photoperiod (<15.0–15.5 h), the proportion of females with fully developed ovaries is drastically reduced. Contrastingly, increasing photoperiod is closely related to increased reproductive output [45]. In our research, the impact of the photoperiod on fecundity is particularly pronounced in the second generation. In Italy, oviposition begins in mid-July and reaches its peak at the end of July, when the photoperiod is >15 h [30], while the Slovenian second generation peak oviposition is moved to the second half of August, when photoperiod is already <14.5. Therefore, these are to be considered as the main causes of the longer pre-oviposition period and lower fecundity found in Slovenia. Temperature as a critical factor influencing the prolongation of the pre-oviposition period and fecundity of *H. halys* was also pointed out in a study conducted by Govidan et al. [53]. According to Nielsen et al. [54], differences in pre-oviposition period and fecundity could be attributed to genetic differences in the geographic populations, rearing conditions, or differences in the collection date of the specimens used in the study. Scaccini et al. [49], who found that exposure to low temperatures during the winter can increase the longevity of *H. halys* but reduces the fecundity of females, gave an additional explanation for the lower fecundity obtained in our study.

Here we present data on development time obtained at natural fluctuating temperatures under outdoor conditions. Over a 3-year period, developmental time and average temperature during the development period were calculated for all adults of the first and second generation. In line with the aforementioned studies, the development time from egg to adult was closely linked with temperature. In general, the development time decreased with increasing temperatures. The shortest development time recorded was 38 days, at an average temperature of 25 °C during the development period. This result is fully consistent with the findings of the laboratory experiments conducted by Nielsen et al. [54], who suggested 25 °C as the optimal temperature for the development of *H. halys*, based on the shortest development time and the highest survival obtained in the experiments. The opposite trend was observed in specimens that completed development in autumn. With decreasing temperatures, the development time was extended. The last specimens that were able to complete development, hatched in late August and molted into adults in early November. At the average temperature 16.8 °C measured during this period, it takes 67 to 81 days to complete development. The lowest temperature at which *H. halys* was able to complete development, as observed in our study (16.8 °C), is consistent with findings of Nielsen et al. [54], Haye et al. [29] and Govidan et al. [53], who suggested that the lowest developmental thresholds for adults is 17 °C. 

Considering the minimum temperature threshold of 12.2 °C [29], development from egg to adult requires 530 DD for the first generation and 545 DD for the second generation. The development of *H. halys* Slovenian populations is comparable to the Swiss population (588.24 DD), to those of the United States where 538 DD are needed with a minimum temperature threshold of 14.14 °C [54] and similar to the Caucasian population, where the pre-adult development requires 530 to 590 DD at the lower developmental thresholds of 13.3 °C [45].

Since *H. halys* was first found in Slovenia in 2017, increasing populations and crop damage have been observed [23]. The present research is largely consistent with the current situation in the field. The realized net reproductive rates (R_0_) for the Slovenian populations in 2019 were 14.8 for the first generation and 5.6 for the second generation. Since the research was performed in outdoor conditions, it provided a clear view of temperature-dependent development and population growth. Considering that temperature directly affects insect population dynamics by changing rates of development, reproduction and mortality [55], the biological parameters of *H. halys* are expected to vary within years. Although our study did not include limiting factors, such as the contribution of natural enemy-induced mortality, recent findings suggest that biological control is expected to play an important role in pest suppression over the coming years.

Since *H. halys* has only recently invaded the Slovenian territory, there is still lack of natural enemies that can effectively limit its population growth and spread. So far, the most promising natural enemies of *H. halys* found in Slovenia are the egg parasitoids *Anastatus bifasciatus* (Geoffroy) and *Trissolcus mitsukurii* (Ashmead) [56]. The first is a native species widely distributed in Europe, with a moderate level of *H. halys* suppression. *Trissolcus mitsukurii* is non-native parasitoid and currently has limited distribution in Europe, but it has a high potential to expand its range globally and help mitigate the negative effects of *H. halys* in invaded areas [57].

The present study confirmed that Slovenia has favorable climatic conditions for development of two generations per year, which causes high population growth and increases the risk of damage to agricultural production. For the above reasons, the management of *H. halys* remains a very challenging task. Many researchers have worked hard over the past decade to provide alternative management tactics that are effective and environmentally acceptable as well. In this 3-year research study, key phenological data and information on the biological parameters of *H. halys* were obtained, which helps to optimize control measures and adapt them, especially in relation to the most vulnerable life stages of the pest. Regardless of the control method used, its effectiveness depends on an optimal timing and this must be taken into consideration both when applying pesticides or when releasing natural enemies. Knowledge of the phenology of local populations makes it possible to predict the occurrence of particular development stages of in the field. The results obtained in this work could also be applied in the development of forecasting models as important decision-making tools, contributing to a more efficient management of this globally invasive species.

## Figures and Tables

**Table 1:** Fertility and mortality data of *Halyomorpha halys* overwintering generation kept at outdoor conditions at Nova Gorica (western Slovenia) in 2019.

Oviposition Period	Total N^o^. of Eggs Laid Per Week (e_x_)	Proportion of Total N^o^. Eggs Laid (%) (e_x_ × 100/E)	N^o^. of N2 Followed	% Mortality at Different Stages						% Total Mortality
				Eggs	N1	N2	N3	N4	N5	
27 May–2 June	49	2.8	30	18.37	17.50	16.67	8.00	0.00	0.00	46.94
3–6 June	215	12.5	40	11.82	11.34	20.00	6.25	6.67	7.69	25.07
10–16 June	264	15.3	40	20.79	15.00	17.50	15.15	7.14	19.23	39.87
17–23 June	292	16.9	40	16.36	26.37	30.00	17.86	4.35	4.55	44.93
24–30 June	256	14.8	40	18.18	30.00	32.50	11.11	0.00	8.33	49.76
1–7 July	163	9.5	40	14.29	8.33	20.00	6.25	3.33	13.79	30.63
8–14 July	136	7.9	40	11.83	14.63	22.50	12.90	11.11	33.33	42.38
15–21 July	106	6.1	40	14.15	17.58	30.00	39.29	0.00	0.00	50.94
22–28 July	78	4.5	40	12.82	5.88	15.00	0.00	0.00	5.88	28.21
29 July–4 August	57	3.3	30	15.79	20.83	10.00	0.00	3.70	3.85	42.11
5–11 August	54	3.1	30	40.74	12.50	13.33	3.85	0.00	0.00	57.41
12–18 August	26	1.5	18	30.77	0.00	55.56	12.50	0.00	14.29	76.92
19–25 August	28	1.6	24	14.29	0.00	8.33	22.73	29.41	50.00	78.57
Mean (SE)				18.48 ± 2.32	13.84 ± 2.51	22.41 ± 3.47	11.99 ± 2.93	5.06 ± 2.37	12.38 ± 4.07	
Total (E)	1724									

“ex” indicates total number of eggs laid per week.

**Table 2:** Fertility and mortality data of *Halyomorpha halys* summer generation kept at outdoor conditions at Nova Gorica (western Slovenia) in 2019.

Oviposition Period	Total No. of Eggs Laid Per Week (e_x_)	Proportion of Total No. Eggs Laid (%) (e_x_ × 100/E)	N2 Followed	% Mortality at Different Stages						% Total Mortality
				Eggs	N1	N2	N3	N4	N5	
29 July–4 August	83	10.9	30	18.07	7.35	20.00	0.00	0.00	0.00	31.33
5–11 August	231	30.4	40	7.27	24.51	35.00	11.54	13.04	5.00	37.79
12–18 August	241	31.7	40	19.44	18.39	22.50	16.13	19.23	4.76	40.48
19–25 August	110	14.5	40	9.09	4.00	20.00	21.88	8.00	56.52	33.64
26 August–1 September	68	8.9	40	7.35	6.35	10.00	14.29	0.00	16.67	20.59
2–8 September	27	3.6	16	25.93	20.00	12.50	100.00			100.00
Mean (SE)				14.53 ± 2.69	13.43 ± 3.50	20.00 ± 3.59	27.30 ± 14.84	8.05 ± 3.74	16.59 ± 10.35	
Total (E)	760									

**Table 3:** Mean developmental time egg-to-adult for *Halyomorpha halys* at outdoor temperatures in the period 2019–2021 at Nova Gorica (western Slovenia).

Year	The Length of Pre-Oviposition Period (Days)				Egg-To-Adult Development Time (Days)			
	Overwintering Generation	Mean Temp. (°C)	Summer Generation	Mean Temp. (°C)	Overwintering Generation	Mean Temp. (°C)	Summer Generation	Mean Temp. (°C)
2019	40.80 ± 1.59	13.6	17.30 ± 0.42	24.8	48.17 ± 0.42	23.8	59.23 ± 1.24	19.4
2020	33.20 ± 2.23	15.9	18.60 ± 0.58	23.8	59.33 ± 0.60	22.7	53.95 ± 0.90	20.8
2021	44.30 ± 1.14	13.3	14.00 ± 1.11	24.7	48.93 ± 0.59	24.1	54.48 ± 0.74	21.3
Mean	39.43 ± 3.28		16.63 ± 1.37		52.14 ± 3.60		55.89 ± 1.68	

**Table 4:** Total degree days (DD_12.2_) required for *Halyomorpha halys* development at outdoor temperatures, Nova Gorica (western Slovenia); 2019–2021.

Year	Pre-Oviposition Period (DD_12.2_)		Egg-To-Adult Development (DD_12.2_)	
	Overwintering Generation	Summer Generation	Overwintering Generation	Summer Generation
2019	113.1	205.40	553.3	523.5
2020	130.2	183.9	510.5	534.2
2021	113.0	147.7	528.8	577.8
Mean	118.77 ± 7.72	179.00 ± 16.84	530.87 ± 12.40	545.17 ± 16.61

**Table 5:** Age specific life table for overwintering generation of *Halyomorpha halys* kept at outdoor conditions in Nova Gorica (western Slovenia) in 2019.

Stage	l_x_	d_x_	Apparent Mortality q_x_ (d_x_/l_x_)	Real Mortality r_x_ (d_x_/l_0_)	k Value (−log (1 − q_x_))	% of Generational Mortality (100 k_x_/K_s_)
Egg	1724.00	291.00	0.1688	0.1688	0.0803	19.66
N1	1433.00	250.30	0.1747	0.1452	0.0834	20.42
N2	1182.70	272.10	0.2301	0.1578	0.1135	27.81
N3	910.60	110.49	0.1213	0.0641	0.0562	13.76
N4	800.11	38.93	0.0487	0.0226	0.0217	5.31
N5	761.17	87.84	0.1154	0.0510	0.0533	13.04
New generation adults	673.33					
Sex ratio	0.5					
Adult females	336.67					
Realized fecundity	76.0					
Realized F1 progeny	25,586.92					
R_0_	14.84					
Total mortality	60.94%				Ks = 0.4084	

**Table 6:** Age-specific life table for summer generation of *Halyomorpha halys* kept at outdoor conditions in Nova Gorica (western Slovenia) in 2019.

Stage	l_x_	d_x_	Apparent Mortality qx (d_x/_l_x_)	Real Mortality r_x_ (d_x_/l_0_)	k Value (−log (1 − q_x_))	% of Generational Mortality (100 k_x_/K_s_)
Egg	760.00	100.66	0.1324	0.1324	0.0617	6.21
N1	659.34	105.20	0.1596	0.1384	0.0755	7.59
N2	554.14	131.94	0.2381	0.1736	0.1181	11.88
N3	422.19	70.32	0.1666	0.0925	0.0791	7.96
N4	351.87	36.73	0.1044	0.0483	0.0479	4.82
N5	315.14	46.79	0.1485	0.0616	0.0698	7.02
Diapausing adults	268.35	211.67	0.7129	0.2785	0.5420	54.52
Overwintering adults	77.04					
Sex ratio	0.5					
Adult females	38.52					
Realized fecundity	111,3					
Realized F1 progeny	4287.47					
R_0_	5.64					
Total mortality	89.86 %				Ks = 0.9941	

## Data Availability

Data supporting results presented in this study are available on request from the corresponding author (M.R.)
